# A fast, reliable and sample-sparing method to identify fibre types of single muscle fibres

**DOI:** 10.1038/s41598-019-42168-z

**Published:** 2019-04-24

**Authors:** Danny Christiansen, Martin J. MacInnis, Evelyn Zacharewicz, Hongyang Xu, Barnaby P. Frankish, Robyn M. Murphy

**Affiliations:** 1 Department of Biochemistry and Genetics, La Trobe Institute for Molecular Science, La Trobe University, Melbourne, Victoria 3086 Australia; 2 Institute for Health and Sport (IHES), Victoria University, Melbourne, Australia; 3 Faculty of Kinesiology, University of Calgary, Calgary, Canada

**Keywords:** Immunoblotting, Physiology

## Abstract

Many skeletal muscle proteins are present in a cell-specific or fibre-type dependent manner. Stimuli such as exercise, aging, and disease have been reported to result in fibre-specific responses in protein abundances. Thus, fibre-type-specific determination of the content of specific proteins provides enhanced mechanistic understanding of muscle physiology and biochemistry compared with typically performed whole-muscle homogenate analyses. This analysis, however, is laborious and typically not performed. We present a novel dot blotting method for easy and rapid determination of skeletal muscle fibre type based on myosin heavy chain (MHC) isoform presence. Requiring only small amounts of starting muscle tissue (*i*.*e*., 2–10 mg wet weight), muscle fibre type is determined in one-tenth of a 1–3-mm fibre segment, with the remainder of each segment pooled with fibre segments of the same type (I or II) for subsequent protein quantification by western blotting. This method, which we validated using standard western blotting, is much simpler and cheaper than previous methods and is adaptable for laboratories routinely performing biochemical analyses. Use of dot blotting for fibre typing will facilitate investigations of fibre-specific responses to diverse stimuli, which will advance our understanding of skeletal muscle physiology and biochemistry.

## Introduction

Skeletal muscle is a heterogeneous and dynamic tissue with respect to fibre type. In humans, skeletal muscle fibres are characterised according to the presence of myosin heavy chain (MHC) isoforms as type I, IIa, and IIx, but I/IIa and IIa/IIx hybrid fibres are also present^1–5^. Fibre types have different biochemical and physiological properties. In comparison to type II (“fast twitch”) fibres, type I (“slow-twitch”) fibres have a slower rate of force production and sarcoplasmic reticulum (SR) Ca^2+^ kinetics, altered glycogen utilization, possess more mitochondria, and are more resistant to fatigue^6–12^. Reflecting their distinct physiology and biochemistry, protein content and metabolic regulation differ across skeletal muscle fibre types. In large muscles, fibres are recruited according to the force output required (type I before type II), and the proportion of each skeletal muscle fibre type is associated with the function of a particular muscle and the characteristics of the individual (*e*.*g*., genetics, fitness, and age)^13^. Thus, fibre type-specific protein analyses are critical for a more mechanistic understanding of skeletal muscle physiology.

The many published studies that have addressed fibre-specific aspects of skeletal muscle physiology, metabolism, and biochemistry required tedious, costly, and time-consuming sample collection and analyses^6–11,14^. Previously, our research group presented a method for analysing protein content in fibre segments (~1–3-mm segments) of freeze-dried skeletal muscle fibres^15^. Using this method, which is predicated on sensitive Western blotting, it is possible to simultaneously determine the fibre type and relative abundance of proteins of interest in an individual fibre segment. This method, or modifications of it, has been used successfully to study skeletal muscle cell physiology at the fibre type level^16–18^ as well as skeletal muscle fibre-specific responses to acute^19–21^ and chronic exercise^22,23^, aging^17,24^ and disease^25^. Whilst this method drastically reduced the time required for fibre type-specific analysis compared to earlier investigations^6–11^, it remains somewhat hindered by the time and costs associated with separately analysing protein content in 100 s to 1000 s of individual muscle fibre samples for a single study.

In this manuscript, we describe an advance in the analysis of fibre-type specific protein content in skeletal muscle biopsy samples. Briefly, fibre segments were collected, and the equivalent of ~1/10 of each fibre segment was dot blotted for detection of MHC IIa and proteins. According to this detection, fibre segments were pooled to form samples of type I and type IIa fibre segments for Western blot analysis^20,25^. The main advantages of this method are that it requires very little sample (*i*.*e*., ~2–10 mg wet-weight tissue is sufficient for single fibre analyses, compared to 20–60 mg used previously^15,19,26^), is simple and quick to perform and drastically reduces the costs (~40-fold) associated with fibre type-specific muscle analyses. While the presented method is advantageous in detecting type I and IIa fibres (and IIx fibres through a process of elimination using antibodies specific to other MHC isoforms) compared to existing methods, it cannot be used to detect hybrid IIa/IIx fibres at present due to cross-reactivity of the IIx antibody with other MHC isoforms.

## Results

### Comparison of dot blotting and Western blotting for fibre type determination

**Figure 1:**
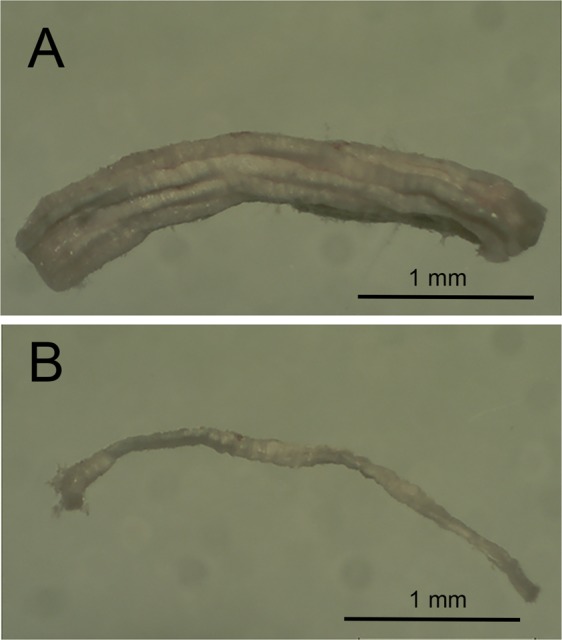
Representative images of a freeze-dried muscle fibre bundle (**A**) and a fibre (**B**) at 20x magnification. Scale bar as indicated.

**Figure 2:**
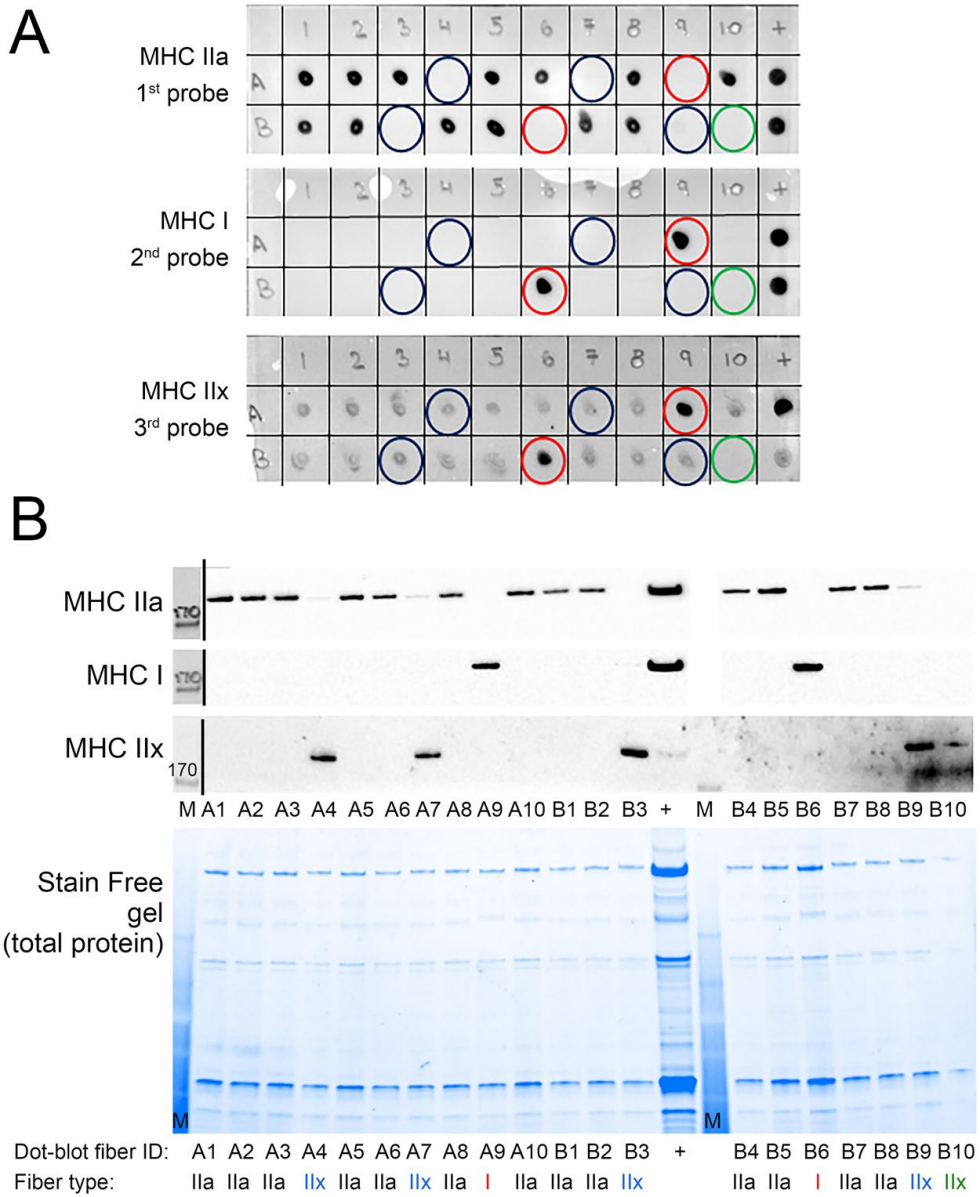
Fibre segments from human vastus lateralis muscle identified for myosin heavy chain (MHC) isoforms by dot blotting and Western blotting (**A**) 1/10 of each fibre segment was dot blotted (positions A1-10, B1-10), along with control muscle samples (+lane). Based on MHC isoform expression, fibre segments were characterised as type I (red circles), IIx (blue circles), or IIa (no circle). There was one dot/sample that remained negative for all three probes (green circles). (**B**) Fibre type of fibre segments from positions A1-10 and B1-10, was also determined by Western blotting using 1/5 of each fibre segment sample. The 170 kDa molecular marker can be seen for each blot (lanes labelled M). A single Stain Free gel is shown as a representation of the gels, and is indicative of the total protein loaded. Lanes are numbered according to the positions on the dot blots in (A) and below are indicated the assigned fibre type. Thirteen type IIa (black text), two type I (red text), and four type IIx fibre segments (blue text) were positively identified. Fibre type of the fibre segment at position B10 could not be determined using dot blotting, but was characterised as a type IIx fibre using Western blotting (green text).

**Figure 3:**
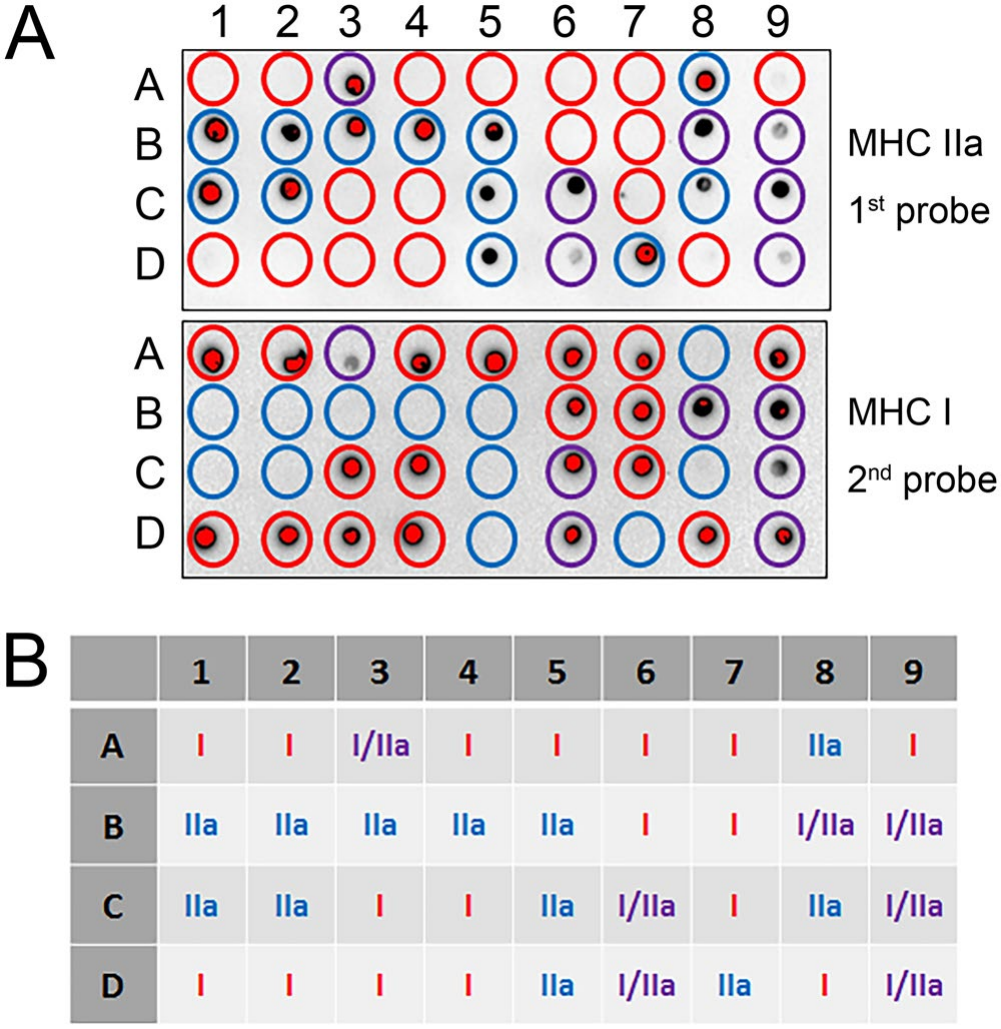
Fibre segments identified as hybrid (purple circles) for myosin heavy chain isoforms by dot blotting. (**A**) Fibre segments were identified as type IIa (circled in blue), type I (circled in red), or as hybrid (I/IIa; circled in purple). Note red dots are present as the membrane was purposely overexposed to maximise the ability to observe dots of weaker intensity. Classification of each fibre analysed shown in (**B**).

The fibre types of the fibre segments (Fig. 1) were identified by dot blotting the equivalent of 1/10 of each fibre segment sample (i.e. 1 µL) to PVDF membrane and sequentially probing with antibodies against MHC IIa, MHC I, and MHC IIx. The first probe identified 13 of the 20 fibre segments as being positive for MHC IIa, with the two whole-muscle control samples also positive for MHC IIa (Fig. 2A, top). Those fibre segments that did not contain MHC IIa have been indicated with a circle, and following the second probe with MHC I, two of those fibre segments were positive (Fig. 2A, middle, red circles showing the MHC I positive fibres). The final probe showed that most fibre segments were immunoreactive with MHC IIx antibody (Fig. 2A, bottom). Thus, this antibody is clearly unspecific when used in dot blotting (i.e., it detects multiple MHC isoforms). It is clear, however, that four of the blue-circled fibre segments had not stained previously for MHC IIa or MHC I, but now stained positive for the first time using the MHC IIx antibody and these fibre segments were therefore identified as type IIx fibres, through a process of elimination (blue circles, Fig. 2A). Hybrid I/IIa fibre segments can clearly be detected, as seen in a further subset of fibre segments which reacted with MHC IIa and/or MHC I antibodies, identified with blue (MHC IIa), red (MHC I) and purple (MHC I/IIa) circles (Fig. 3).

To validate the dot-blotting results, 1/5 of each of the same samples were run on Criterion Stain Free SDS-PAGE gels. Following transfer to membrane, the membrane was probed with the same MHC antibodies as used for dot blotting. In all cases, fibre segments identified as type I, IIa or IIx using dot blotting (Fig. 2A) showed the same identification using Western blotting (Fig. 2B). The fibre segment in position B10 was not identified with dot blotting, but with Western blotting was revealed to be a type IIx fibre segment. The lack of identification using dot blotting was likely due to the small size of that fibre segment, which was confirmed by the Stain Free gel image of fibre total protein content (*e*.*g*., compare lanes of fibre segments B9 and B10 that demonstrate the much lower total protein and hence fibre segment size of B10). Thus, the concordance between the two techniques was 100%.

### Fibre type dependence of protein abundance

**Figure 4:**
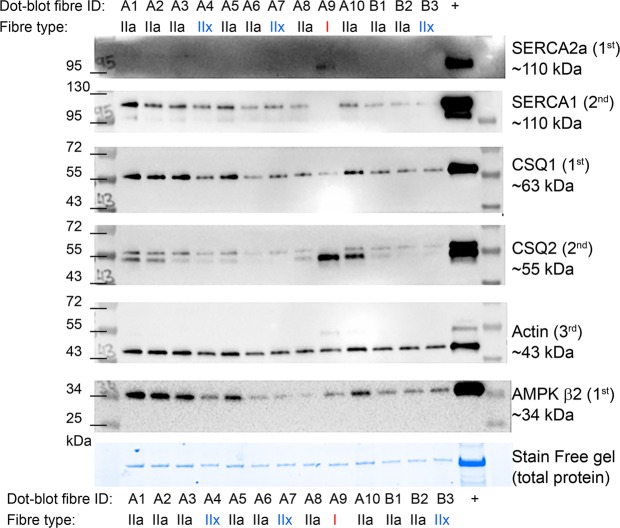
Fibre-type specific expression of various proteins in fibre segments. The 14 fibre segments shown in the Western blot in Fig. 2B were probed for SERCA1, CSQ1, and AMPK β2 (1^st^ probes in the respective regions of the membrane) and SERCA2a and CSQ2 (2^nd^ probes), and Actin (3^rd^ probe), with no stripping of membranes between probes. As seen, the fibre type determined by dot blotting of each fibre segment (Dot-blot fiber ID) corresponds to the expected fibre-specific expression of these proteins, as determined by western blotting. Note that the CSQ antibody detected both CSQ1 (upper band) and CSQ2 (lower band). As in Fig. 2, the fibre type identification is indicated above the blots and below the Stain Free gel. Sizes of molecular weight markers are indicated on the left of each blot, whereas sizes for the proteins of interest are on the right.

Following fibre type verification by Western blotting, the membranes were probed for SR Ca^2+^-ATPase isoforms 1 (SERCA1) and 2 (SERCA2a), the calcium-binding protein, calsequestrin, isoforms 1 (CSQ1) and 2 (CSQ2), the contractile protein, Actin and the beta 2 subunit of the energy-sensing protein, AMP kinase (AMPKβ2) (Figs 4 and S1). SERCA1 and SERCA2a were only present in type II or type I fibres, respectively. CSQ content was variable between the fibre types, although CSQ1, the fast (type II) isoform, was least abundant in type I fibres, which showed the highest expression of CSQ2, the slow (type I) isoform. These provide some validation that the MHC antibodies are specific for fast and slow-isoforms, as previously shown^27,28^. Actin was similarly abundant in fibres, showing a similar expression to that of the total protein gel, as expected for a contractile protein that is abundant in both fast and slow-twitch muscle fibres. The expression of AMPKβ2 showed the most variability across the fibres, regardless of the MHC isoforms present (Fig. 4).

### Variation and reliability of Western blotting for individual skeletal muscle fibres

**Figure 5:**
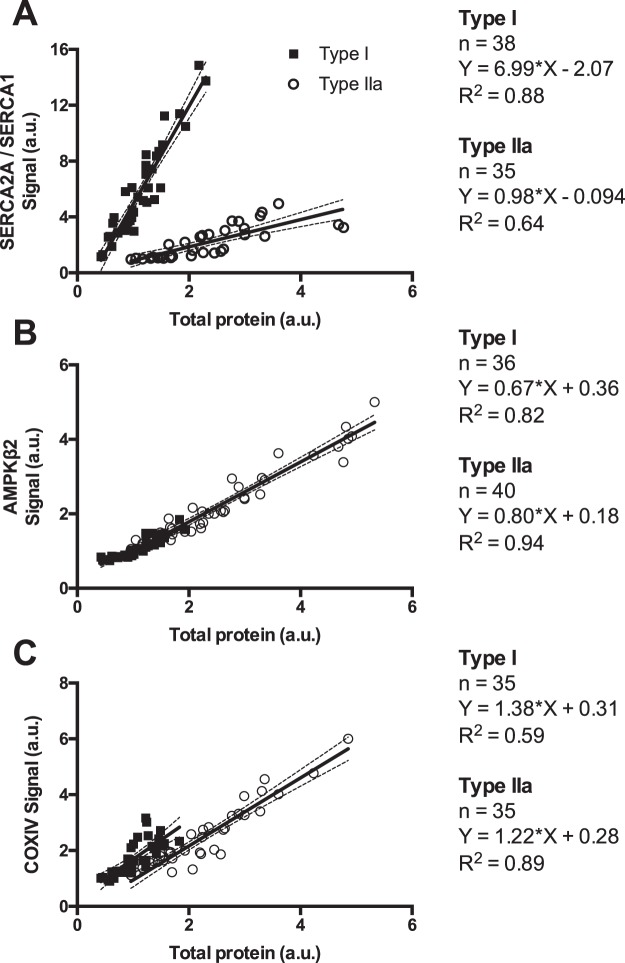
Linear relationships between Western blot signals and total protein content for SERCA2A/SERCA1 (**A**), AMPKβ2 (**B**), and COXIV (**C**) in individual type I (black squares) and type IIa (open circles) human skeletal muscle fibre segments. Sample sizes, equations for linear relationships, and coefficients of determination are shown for each fibre type. The solid line indicates the line of regression, and the dashed lines indicate the 95% confidence interval of each linear regression.

We sought to quantify the variability in total protein content, as well as Western blot signal and normalised content of proteins of interest across individual type I and IIa skeletal muscle fibres collected from a single human muscle biopsy, to ensure only intra-individual biological variability would be present. As shown by the CV and IQR in Supplementary Table S1, there was considerable variability in the total protein content of type I and IIa fibres (i.e., the amount of protein in each 4-µL aliquot) and the Western blot signals for the proteins of interest. This was as expected, because fibre size was purposely not heavily considered when fibres were being isolated, simply because fibre size is weighted heavily on fibre radius (r, typically 30–40 μm) and less on fibre length (l, typically 1–3 mm, where volume = πr^2^ l) and radius (as seen by the fibre diameter) cannot be accurately assessed using a dissecting microscope. This aside, ICC values were good to excellent, and typical errors (as a CV) were mostly below 20%, suggesting that these measures were reliable. Further, the subsequent analyses accounted for variability in fibre size, whereby normalising the Western blot signals to the total protein content (Supplementary Table S1) decreased the CVs and IQR for normalised protein contents considerably, owing to the moderate to strong linear relationships between total protein content and Western blot signal (Fig. 5). While the ICC values were lower for normalised protein content than raw values (Table 1), they were still moderate to good, and the typical errors (as a CV, Table 1) were mostly unchanged, demonstrating good reliability of the technique.

**Table 1:** Measures of reliability for total protein content, Western blot signals, and normalised protein content for proteins of interest in individual type I and type IIa human skeletal muscle fibre segments.

Fibre type	Protein^a^	Protein content or Western blot signal			Normalised protein content		
		Typical Error, a.u.^b^	Typical Error as CV, %	ICC^c^	Typical Error, a.u.^b^	Typical Error as CV, %	ICC^c^
Type I	Total	0.12 (0.10–0.15)	11.5 (9.3–15.0)	0.97 (0.94–0.98)	—	—	—
Type I	SERCA2A	0.85 (0.70–1.10)	22.4 (18.0–29.7)	0.97 (0.95–0.98)	0.78 (0.64–1.00)	20.3 (16.4–26.8)	0.70 (0.50–0.83)
Type I	AMPKβ2	0.25 (0.21–0.33)	16.2 (13.1–21.3)	0.87 (0.77–0.93)	0.20 (0.16–0.25)	18.2 (14.7–24.0)	0.50 (0.23–0.70)
Type I	COXIV	0.64 (0.52–0.82)	19.5 (15.7–25.7)	0.81 (0.68–0.90)	0.30 (0.25–0.39)	15.3 (12.3–20)	0.67 (0.46–0.81)
Type IIa	Total	0.26 (0.21–0.33)	8.0 (6.5–10.4)	0.96 (0.93–0.98)	—	—	—
Type IIa	SERCA1	0.59 (0.48–0.76)	11.4 (9.3–14.9)	0.90 (0.82–0.94)	0.09 (0.08–0.12)	9.6 (7.8–12.5)	0.87 (0.78–0.93)
Type IIa	AMPKβ2	0.40 (0.33–0.51)	15.4 (12.5–20.2)	0.86 (0.75–0.92)	0.09 (0.08–0.12)	10.7 (8.7–14.0)	0.56 (0.31–0.74)
Type IIa	COXIV	0.83 (0.68–1.06)	16.8 (13.5–22.0)	0.87 (0.77–0.93)	0.13 (0.11–0.17)	11.2 (9.1–14.6)	0.82 (0.68–0.90)

^a^For **“**Total,” data reflect the total amount of protein loaded per lane, as measured by UV exposure of the Criterion gel; For the proteins of interest, data reflect the non-normalised Western blot signal or the normalised protein content.

^b^Arbitrary units (a.u.) are derived from a 4-point calibration curve of mixed-muscle homogenate that was loaded on every gel.

^c^Intraclass correlation coefficient (ICC) ranges were good to excellent for protein content or Western blot signal (i.e., >0.7) and moderate to good for normalised protein content (i.e., > 0.5); however, note that the reduction in ICC with normalisation is related to the overall reduction in variance in the dataset (see text).

*n* = 40 for all datasets; reliability statistics are based on comparisons of the sets of two replicates.

Values in parentheses are 95% confidence intervals.

### Validation of pooling muscle fibres

Given the variability of normalised protein content in fibres of the same type (Supplementary Table S1), we proceeded to investigate the theoretical variability associated with the pooling of different numbers of fibres from the same skeletal muscle biopsy. We performed this analysis through a simulation that executed each sampling protocol (i.e., sampling 1 to 40 fibres) 1000 times, using the datasets generated from the 40 type I and type IIa fibres. As shown in Table 2, the width of the 95% confidence intervals associated with pooling different numbers of type I and IIa fibres decreased with increasing numbers of pooled fibres. Breakpoint values ranged from 3 to 9 fibres, after which all slopes were relatively small (i.e., <0.03 a.u. reduction in the 95% confidence interval width per additional fibre added to the pooled sample).

**Table 2:** The 95% confidence interval widths for normalised protein content estimates derived from a simulated “pooling” of different numbers of type I and type IIa human skeletal muscle fibre segments.

Protein	Fibre type	Mean, a.u.^a^	95% Confidence interval width, a.u. (% of mean)^a^									Break point, a.u. (SE)^a^	Slope, a.u. (SE)^a,b^
			Number of fibres pooled										
			1	5	10	15	20	25	30	35	40		
SERCA2A	I	4.97	4.12 (83)	2.25 (45)	1.56 (31)	1.20 (24)	1.12 (23)	1.03 (21)	0.86 (17)	0.83 (17)	0.82 (17)	6.6 (0.30)	−0.028 (0.002)
SERCA1	IIa	0.94	0.72 (76)	0.45 (48)	0.29 (31)	0.24 (26)	0.21 (22)	0.19 (20)	0.18 (19)	0.17 (18)	0.15 (16)	8.5 (0.33)	−0.005 (0.0004)
AMPKβ2	I	1.08	0.91 (85)	0.42 (39)	0.28 (26)	0.23 (21)	0.20 (19)	0.19 (17)	0.17 (16)	0.16 (14)	0.14 (13)	4.6 (0.24)	−0.006 (0.0005)
	IIa	0.88	0.53 (60)	0.21 (24)	0.15 (17)	0.13 (14)	0.10 (12)	0.10 (11)	0.08 (9)	0.08 (10)	0.08 (9)	3.5 (0.20)	−0.003 (0.0003)
COXIV	I	1.88	1.87 (94)	0.84 (43)	0.60 (30)	0.48 (24)	0.42 (21)	0.36 (18)	0.34 (17)	0.31 (16)	0.29 (15)	4.4 (0.24)	−0.012 (0.001)
	IIa	1.15	1.30 (113)	0.51 (44)	0.35 (30)	0.30 (26)	0.25 (22)	0.22 (19)	0.22 (19)	0.18 (16)	0.18 (15)	3.4 (0.21)	−0.008 (0.0009)

^a^Arbitrary units (a.u.) are derived from a 4-point calibration curve of mixed-muscle homogenate that was loaded on every gel.

^b^The slope of the relationship between the 95% confidence interval width and the number of fibre segments pooled after the breakpoint.

## Discussion

This manuscript presents an advance in methodology for fibre type identification of skeletal muscle fibres that can easily be introduced into laboratories studying skeletal muscle physiology and biochemistry. The work expands previous progress made in the ability to analyse proteins in segments of single skeletal muscle fibres^15^. By following our prescribed methodology, the ability to collect and analyse skeletal muscle samples for relative, quantitative measurements of proteins in specific fibre types is very accessible. This new method saves considerable time and money, because no gels or transfer step are needed and a less sensitive chemiluminescence reagent can be used. We estimate that this method reduces the cost to fibre type 50 fibre segment by ~40 fold (AUD $64 vs $1.40).

Our first key finding is that qualitative determination of fibre type of an isolated muscle fibre can be performed using dot blotting of 1/10 of a 1–3 mm fibre segment dissected from a human muscle biopsy. The validity of dot blotting was demonstrated by Western blotting of another portion of the same fibre segment and detecting MHC isoforms (Fig. 2), as performed frequently by us^15–19,22,24,29^ and others^20,25^. Thus, whilst either technique could be used to determine the fibre type of individual muscle fibre segments, the dot blotting procedure is much quicker and simpler to perform. It allows 100 s of individual fibres to be fibre typed in one day in contrast to 10 s of fibres over two or more days using conventional Western blotting, Furthermore, dot blotting is dramatically cheaper because no gels or transfer steps are required and a less sensitive chemiluminescence reagent can be used. Previously, protein densitometry analyses had to be performed separately for each fibre segment before averaging the data, which markedly increased the time required for fibre-specific analysis. With dot blotting, rapid detection of fibre type proceeds pooling of fibres, and proteins can then be quantified at the pooled-fibre level using modified Western blotting^20,21,25^. While there might be potential in the presented method to determine an individual’s fibre-type distribution of a given muscle biopsied, this would require a much larger number of fibre segments be dissected and that there be no bias in selection of fibres from the muscle biopsy. Thus, in its presented form, we do not recommend dot blotting for assessing fibre-type distribution in a muscle biopsy.

We demonstrate the ability to identify MHC I/IIa hybrid fibres using dot blotting (Fig. 3). In this regard, when considering if a given fibre is a hybrid fibre, we suggest that having <5% of a given MHC isoform will not greatly alter speed of contraction of a muscle fibre over the predominant MHC present, and consequently, a threshold of <5% could be used as cut-off to determine whether a fibre should be characterized as a hybrid. Further, we were able to identify single fibres expressing only the MHC IIx isoform with the 6H1 (MHC IIx) antibody using Western blotting. In dot blotting, however, this antibody did cross-react with other MHC isoforms. Despite this cross-reactivity, an unequivocal determination can be made for MHC IIx fibres through a process of elimination: although all samples were positive for MHC IIx, we demonstrated, by Western blotting, that those fibres that were negative for MHC I and MHC IIa but positive for MHC IIx were indeed MHC IIx fibres segments (Fig. 2A). We note that it is not possible to detect hybrid type IIa/IIx fibres, due to cross-reactivity with other MHC isoforms of the antibody used to detect MHC IIx. Indeed, sequence identity of MHC I (Myosin-1[Homo sapiens], Accession NP_005954) and IIx (Myosin Heavy Chain IIX/D [Homo sapiens], Accession AAD29951) revealed only a single amino acid difference between the two proteins (Protein Blast analyses, PubMed). With IIa/IIx fibres possibly accounting for 3–10% of the total skeletal muscle fibre pool^30^ and considering the relative proportion of these hybrid fibres within the total fibre pool may increase with certain disease states or in muscle obtained from old individuals, the cross-reactivity of this antibody is currently a limitation of the presented method.

To clarify the number of fibres required for valid quantification of fibre type-specific protein content, we addressed three potential issues: (i) the variability in protein content in type I and type IIa skeletal muscle fibres from a single human muscle biopsy; (ii) the relationships between Western blot signals and total protein content; and (iii) the variability associated with different numbers of pooled fibres. As we have previously highlighted^31^, the variability of normalised protein content within a given fibre type from a given individual is not trivial (CVs between ~15–25% in the present experiment). This variation was much lower than the variation in total protein content and Western blot signal (generally >50%), which, as stated above, was as expected because fibres of different lengths and diameters were indiscriminately sampled. Due to the moderate to strong linear relationships between total protein content and Western blot signal, using calibration curves and normalising to total protein content accounted for these differences markedly. This finding means there is no issue in loading different amounts of protein (i.e., different size fibres) and obtaining accurate results. Further, this outcome appears independent of protein size, as similar ICC values were obtained for all the investigated proteins, despite large differences in protein size (e.g., Fig. 4). Note that ICC values were lower for normalised protein content than for total protein content or the Western blot signal (Table 1), but this finding is due to the reduction in variance that occurs with normalisation^32^. Typical errors (as a CV) were unaffected by normalisation and remained below 20%.

Finally, we demonstrate that breakpoints in the 95% confidence interval widths occurred between 3 and 9 fibres. While the breakpoint does not necessarily indicate the correct number of fibres to pool, diminishing returns with respect to the accuracy of the pooled fibre mean were apparent when additional fibres were added to the pool. These numbers of fibres are typical of what has been used in the literature^21,23,26^. Further, other defining factors for the number of fibres to be pooled will also be the number of target proteins and the anticipated statistical power. We have demonstrated the ability to measure specific proteins in a typical muscle fibre segment, and also that in order to be quantitative it is most useful to use small sample sizes^33^. As such, the amount of sample corresponding to a pool of 4 fibres (i.e., ~36 µL after dot blotting 1 µL per fibre) will be sufficient in volume to run 4 gels and subsequent western blots, but if there is a greater number of proteins of interest, requiring more gels to be run, then pooling more fibres to provide sufficient sample should be considered during the experimental design of the western blot analyses. Theoretically, adding more than 3–9 fibres to a pooled sample may be necessary for some study designs to accurately represent a total muscle fibre pool. For example, if statistical power is low (e.g., a small difference between groups, and a between-subject design is used), then more fibres will be needed per pooled fibre-type sample to reduce experimental noise. In contrast, if statistical power is large (e.g., a large difference is anticipated between groups, and a within-subject design is used), then relatively few fibres will be needed per pooled fibre-type sample. Further, it should be considered that this minimum number of fibres to pool (3–9) is restricted to the n = 2 healthy humans assessed in the current study. For example, other populations, such as those with diabetes or endurance-trained athletes, might introduce heterogeneity with respect to the biochemical and physiological properties of a given fibre type. This may alter the variability in expression of proteins in different fibre types, and hence the minimum number of fibres required for reproducible protein analysis.

In summary, in skeletal muscle, many proteins involved in a variety of different cellular functions are expressed in a fibre-specific manner. Also, fibre type-specific protein responses to interventions such as a single bout of exercise^19^, exercise training^20,22^, or processes like disease^25^ and aging^24^ have been reported. Such findings highlight the advantage of addressing fibre type heterogeneity in skeletal muscle protein analyses, as well as the necessity to do so. Failure to do so implies that physiologically relevant changes may be overlooked^19,22,24^. Somewhat hindered in the past by the need for highly laborious and time-consuming experiments, the exciting advance presented here provides the means for fibre type-specific studies to be undertaken far more easily in most laboratories. The method presented, and our demonstrated ability to reliably measure the abundance of proteins of varying absolute abundance in groups of only a few fibres, opens the way for improvements in our understanding of how muscle fibre type may play a crucial regulatory role in skeletal muscle physiology.

## Methods

### Ethical approval and muscle biopsy procedure

The skeletal muscle samples (n = 2) were collected as part of a study approved by the Hamilton Integrated Research Ethics Board (Hamilton, Canada: Study approval: 15–357). The study conformed to the Declaration of Helsinki II and participants provided informed consent prior to participation. Separate data from this study have been published^26^. Both participants were males who performed structured exercise 2–3 times per week but were not training for any specific sport. Muscle biopsy samples were collected at rest prior to an exercise training intervention. The participants had an average age of 26.0 (4.2) years, a body mass index of 27.4 (3.0) kg/m^2^, and a maximum oxygen uptake of 49.6 (2.3) mL/kg/min. Briefly, samples were obtained from the *vastus lateralis* muscle under local anaesthesia (1% Xylocaine) using a Bergström needle with suction. The study volunteers were healthy males, non-smokers, and engaged in physical activity several days per week.

### Chemicals

General chemicals were from Sigma (Sydney, Australia) and Western blotting solutions and consumables were from BioRad (Hercules, CA, USA) unless otherwise stated.

### Collection of skeletal muscle fibre segments

A muscle sample was freeze-dried for 48 h, brought to room temperature, and 1–3 mm segments of individual muscle fibres were removed under a microscope using jeweler’s forceps, as described by Murphy^15^ (Fig. 1). The average normalized length of a skeletal muscle fibre in human *m*. *vastus lateralis* is ~6.6 cm^34^, with an optimal fibre length of ~11.1 cm^35^. As such, the 1–3 mm lengths of fibre segments collected from muscle biopsies were a small section of any given muscle fibre. Each fibre segment was added to a 0.6-mL tube containing 10 µL of SDS loading buffer (0.125 M Tris-HCI, 10% glycerol, 4% SDS, 4 M urea, 10% 2-mercaptoethanol and 0.001% bromophenol blue, pH 6.8 diluted 2:1 with 1x Tris-HCl (pH 6.8)). Muscle samples were solubilized by vortexing for 5–10 s and exposing to room temperature for 1–2 h, and were then stored at −80 °C until analysis.

### Dot blotting procedure

A PVDF membrane was activated in 95% ethanol for 15–60 s and then equilibrated for 2 min in transfer buffer (25 mM Tris, 192 mM glycine, pH 8.3 and 20% methanol). The wet membrane was placed on a stack of filter paper (one to two pieces soaked in transfer buffer on top of three dry pieces). Samples were thawed and vortexed, but were not centrifuged to avoid pelleting and hence loss of any of the skeletal muscle protein. Samples were spotted to a specific part of the membrane in aliquots equating to 1/10 of a fibre segment (i.e., 1 µL) using a pipette. An aliquot of whole-muscle crude homogenate was added in triplicate as positive controls for MHC I and IIa positive fibres. After complete absorption of samples, the membrane was placed on top of a dry piece of filter paper to dry for 2–5 min before being reactivated in 95% ethanol for 15–60 s and equilibrated in transfer buffer for 2 min. After three quick washes in Tris-buffered saline-Tween (TBST), the membrane was blocked in 5% non-fat milk in TBST (blocking buffer) for 5 min at room temperature. Following blocking, the membrane was rinsed with TBST and then incubated in MHC IIa antibody (mouse monoclonal IgG, clone A4.74, Developmental Studies Hybridoma Bank [DSHB], 1 in 200 in 1% BSA/PBST) at room temperature for 2 h with gentle rocking. Membranes were washed in blocking buffer and then incubated in goat anti-mouse IgG horse radish peroxidase (HRP) secondary antibody (ThermoFisher Scientific: PIE31430, 1 in 20,000 in blocking buffer) at room temperature for 1 h with rocking. Lastly, membranes were washed in TBST and then exposed to Clarity enhanced chemiluminescence reagent (BioRad, Hercules, CA, USA), imaged (ChemiDoc MP, BioRad), and analysed for signal density (ImageLab 5.2.1, BioRad).

The MHC IIa antibody and its secondary antibody were removed from the membrane with stripping buffer (Pierce, Rockford, IL, USA) prior to incubation of the membrane in MHC I antibody (mouse monoclonal IgM, clone A4.840, DSHB, 1 in 200 in 1% BSA/PBST) and goat anti-mouse IgM secondary antibody (Santa Cruz Biotechnology, TX, USA: sc-2064, 1 in 20,000), using the same procedure as for MHC IIa. In some circumstances, a similar process was repeated using the MHC IIx antibody (mouse monoclonal IgM, clone 6H1 DSHB, 1 in 100 in 1%BSA/PBST) and the goat anti-mouse IgM secondary antibody. Using images of all the membranes, it was possible to determine the fibre type of each sample (I, IIa, I/IIa hybrid, IIx) or if no MHC protein was present, which would indicate unsuccessful collection of a fibre segment (Fig. 2). Whilst stripping of membranes may remove a small quantity of protein sample, it is valid in the current setting for MHC I and MHC IIa, because qualitative, and not quantitative, results are required for fibre typing.

### Confirmation of the dot blotting procedure

Following the method of Murphy^15^, a 2-μL aliquot of each sample that was dot blotted, equating to ~1/5 of a fibre segment, was run on a 4–15% Criterion TGX Stain-Free protein gel (BioRad) at 200 V for 45 min. Total protein on the gel was visualised with UV activation, which involved exposure of the gel to UV light that activates endogenous tryptophan amino acids present in the proteins, and an image similar to a coomassie stained gel captured (StainFree Imager, BioRad). Proteins were then wet-transferred to nitrocellulose membrane at 100 V for 30 min in circulating 4 °C transfer buffer. Proper transfer was visualised with the same UV activation as described above, but images were captured from both the gel and the membrane. Membranes were treated with Miser solution (ThermoFisher Scientific) and placed in blocking buffer for 2 h at room temperature before being subjected to a similar Western blotting protocol as described for the dot blotting procedure, except, other than MHC probes, no stripping of membrane between any of the antibody probes. A fibre segment was considered to not express a given MHC isoform if its abundance was <5% of the density seen in other positive lanes. To demonstrate fibre type-dependent protein abundance, in addition to probing for MHC isoforms in the region of the blot above 170 kDa, typically the lower portions of these membranes were cut into three regions (including markers: 95 and 130 kDa; 40 and 55 kDa; 17, 26 and 35 kDa) probed with antibodies (diluted in 1% BSA/PBST) against SERCA1, (mouse monoclonal, DSHB, CA F2-5D, 1 in 1000) and SERCA2 (Badrilla, A010-2,1 in 5000); CSQ1, (mouse monoclonal, Abcam:ab2824, 1 in 2000) and CSQ2 (rabbit, Abcam:ab3516, 1 in 1000); Actin (rabbit polyclonal, Sigma A-2066, 1 in 300); AMPKβ2, (rabbit monoclonal, Cell Signalling #4150, 1 in 1000). For the determination of the linear relationships between the fibre segments containing MHC I or MHC IIa, the mitochondrial content marker, cytochrome c oxidase subunit IV (COX IV, rabbit polyclonal, Cell Signalling #4844, 1 in 1000) was also used. Secondary antibodies were as above or goat anti-rabbit HRP (ThermoFisher Scientific: PIE31460, 1 in 60,000) where suitable. Images of the membrane were captured with the Chemidoc MP after chemiluminescence and then white light images taken without moving the membrane to allow for merging of markers and chemiluminescent imaging.

### Validation of fibre pooling and protein analyses

Another muscle biopsy was freeze-dried for 48 h and dissected. Individual fibre segments were collected, and a small fraction (1/10) of the samples was used to identify fibre type, using the dot blotting method described above, until 40 type I and 40 type IIa fibre segments were identified. Western blotting was performed on aliquots (4-µL) of these individual fibre segments, as described above. Twenty individual fibre segments of each fibre type, and a calibration curve of mixed muscle homogenate (i.e., 1, 2, 4, and 8 µL), were run in separate lanes on each gel. The total protein content and abundance of SERCA1 (type-IIa specific), SERCA2A (type-I specific), AMPKβ2, and COXIV were determined in each fibre segment sample, as described above, with the membrane cut to allow for minimal probes of given regions. Calibration curves were used to express abundances on the same scale across gels. Normalised protein abundance was calculated by dividing the Western blot signal of each protein of interest by the total protein content of the sample, which has also been expressed relative to its calibration curve. All samples were run in duplicate on separate gels. Consequently, protein quantification was performed in duplicate for each fibre segment sample.

## Statistics

In type I and type IIa fibre segments, *n* = 40 for all datasets. The mean, standard deviation, 95% confidence interval, coefficient of variation (CV), and interquartile range (IQR) were calculated in Excel (MS Office 2013, Microsoft, USA) for the total protein content, as well as both Western blot signal and normalised protein abundance of SERCA1, SERCA2A, AMPKβ2, and COXIV after averaging duplicate values. Normality was tested using the D’Agostino and Pearson omnibus normality test in GraphPad Prism (Ver. 6, GraphPad Software, USA). Using the spreadsheet provided by Hopkins^36^, reliability of the Western blotting protocol was determined by calculating the typical error (in A.U. and as a CV) and intraclass correlation coefficient (ICC) for duplicate measures of the total protein, as well as Western blot signal and normalised abundance of proteins of interest. ICC values <0.5, between 0.5 and 0.75, between 0.75 and 0.9 and >0.9 were considered to represent poor, moderate, good, and excellent reliability, respectively^37^. To determine the relationships between total protein content and Western blot signal for each protein of interest, linear regressions and coefficients of determination (R^2^) were performed in GraphPad Prism. Outliers were removed from the linear regression analyses using the ROUT method, with a Q value of 1% (between 0 to 5 samples per protein; see Fig. 5).

Using the datasets of 40 individual fibre segments to represent a whole skeletal muscle biopsy sample, a fibre pooling simulation was performed *in silico* to establish the accuracy of the mean achieved when different numbers of fibre segments were pooled. Briefly, a script was written in R (r-project.org) that randomly selected (with replacement) a specific number of fibre segments from a given dataset and then calculated the mean normalised protein content of this sample. This procedure was repeated 1000 times for all sample sizes between 1 and 40 fibre segments, and the 95% confidence interval of the mean associated with each sample size was determined. Using the “segmented” package in R, the breakpoint in the 95% confidence interval width was determined to identify the sample size at which adding additional fibre segments to the pool began to have a relatively smaller impact on the accuracy of the mean. These procedures were performed for each protein of interest in each fibre type.

## Supplementary information

Supplementary Data

## Data Availability

All authors adhere to the data availability requirement for publication in Scientific Reports and support materials, data and associated protocols are made promptly available to readers without undue qualifications in material transfer agreements.
