# Supplementary material for: A fast, reliable and sample-sparing method to identify fibre types of single muscle fibres
Source: Sci Rep. 2019 Apr 24;9:6473. doi: 10.1038/s41598-019-42168-z (PMC6482153; doi:10.1038/s41598-019-42168-z)
Supplement: Supplementary file 1 — Supplementary Data [file 41598_2019_42168_MOESM1_ESM.pdf]

Title:

A fast, reliable and sample-sparing method to identify fibre types of single muscle fibres

Authors:

Danny Christiansen[1,2 #], Martin J. MacInnis[1,3 #], Evelyn Zacharewicz[1], Hongyang Xu[1], Barnaby P. Frankish[1], Robyn M. Murphy[1 *]

[#] Contributed equally to this work

Supplementary Figure S1

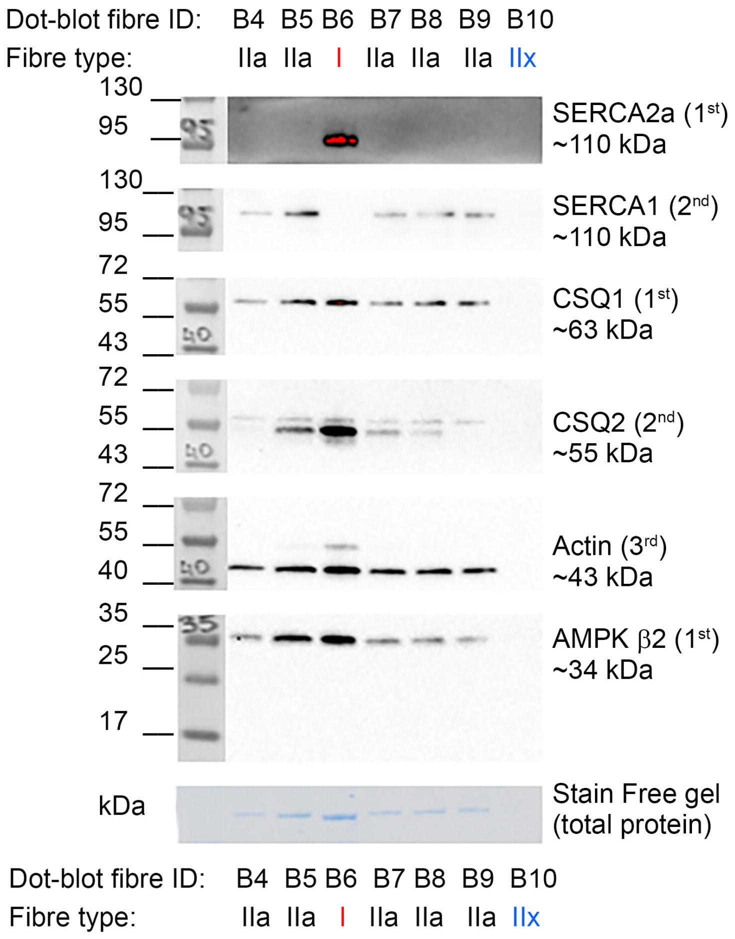

Supplementary Figure 1. *Fibre-type specific expression of various proteins in fibre segments (B3-B10 from Figure 2).*

Similar to Figure 4, but showing the fibres labelled as B3-B10 in Figure 2. These fibres were run on a separate gel to that shown in Figure 4, but details are the same, whereby the 6 fibre segments on the right hand side shown in the Western blot in Figure 2B were probed for SERCA1, CSQ1, and AMPK β2 (1st probes in the respective regions of the membrane) and SERCA2a and CSQ2 (2nd probes), and Actin (3rd probe), with no stripping of membranes between probes. As seen, the fibre type determined by dot blotting of each fibre segment (Dot-blot fiber ID) corresponds to the expected fibre-specific expression of these proteins, as determined by western blotting. Note that the CSQ antibody detected both CSQ1 (upper band) and CSQ2 (lower band). As in Fig. 2, the fibre type identification is indicated above the blots and below the Stain Free gel. Sizes of molecular weight markers are indicated on the left of each blot, whereas sizes for the proteins of interest are on the right.

Supplementary Table 1. Descriptive statistics for total protein content, Western blot signal, and normalised protein content for proteins of interest in individual type I and type IIa human skeletal muscle fibers.

Fiber type	Protein [a]	Protein content / Western blot signal					Normalised protein content				
		Mean, a.u. [b]	SD, a.u. [b]	CV, %	Normality (K2, p)	IQR, a.u. [b]	Mean, a.u. [b]	SD, a.u. [b]	CV, %	Normality (K2, p)	IQR, a.u. [b]
Type I	Total	1.23 (1.03-1.44)	0.66	53.4	24.1; <0.01	0.57	-	-	-	-	-
Type I	SERCA2A	6.60 (5.09-8.11)	0.74	82.9	23.7; <0.01	4.95	4.97 (4.57-5.37)	1.29	25.6	7.67; 0.02	2.04
Type I	AMPKβ2	1.26 (1.06-1.47)	0.67	52.9	36.5; <0.01	0.60	1.08 (1.00-1.15)	0.24	22.2	33.4; < 0.01	0.20
Type I	COXIV	2.16 (1.73-2.59)	1.34	63.8	34.6; <0.01	1.18	1.88 (1.83-2.13)	0.48	24.1	8.42; 0.01	0.64
Type IIa	Total	2.70 (2.30-3.10)	1.29	47.6	4.41; 0.11	1.77	-	-	-	-	-
Type IIa	SERCA1	2.60 (2.05-3.14)	1.76	67.9	9.92; <0.01	2.29	0.94 (0.86-1.02)	0.25	26.4	26.4; < 0.01	0.46
Type IIa	AMPKβ2	2.29 (1.98-2.60)	1.01	44.1	6.41; 0.04	1.41	0.88 (0.84-0.91)	0.12	14.1	4.81; 0.090	0.17
Type IIa	COXIV	3.25 (2.56-3.92)	2.16	66.4	16.3; <0.01	2.13	1.15 (1.06-1.24)	0.28	24.6	15.8; < 0.01	0.16

[a] For "Total," data reflect the total amount of protein loaded per lane, as measured by UV exposure of the Criterion gel; For the proteins of interest, data reflect the non-normalised Western blot signal and the normalised protein content.
[b] The values expressed as arbitrary units (a.u.) were derived from a 4-point calibration curve of mixed-muscle homogenate that was loaded on every gel.
n = 40 for all datasets, with descriptive statistics based on the mean of the two replicates.
Values in parentheses are 95% confidence intervals.
